# Spatiotemporal patterns of amyloid deposition as prognostic markers of Alzheimer’s disease

**DOI:** 10.1007/s00234-025-03781-0

**Published:** 2025-09-30

**Authors:** Shiori Amemiya, Hidemasa Takao, Osamu Abe

**Affiliations:** Department of Radiology, The University of Tokyo, Tokyo, Japan

**Keywords:** Alzheimer's disease, Amyloid beta, Prognostic marker, Positron emission tomography, Spatiotemporal analysis

## Abstract

**Purpose:**

The study aims to characterize the spatiotemporal distribution of amyloid deposition, differentiate between its subtypes, and explore their predictive value for patient cognitive outcomes.

**Methods:**

Amyloid PET data from a prospective consortium study, the Alzheimer’s Disease Neuroimaging Initiative, were used. A spatial independent component analysis revealed regions of amyloid deposition covariation, which served as regions of interest. A subtype and stage inference analysis was then performed to infer spatiotemporal patterns from cross-sectional data. Multinomial logistic regression models evaluated the impacts of demographics and risk factors on subtype assignment and determined the prognostic value of the subtypes for cognitive decline. Longitudinal data were used for validation.

**Results:**

The study included 1,049 participants (466 cognitively normal, 447 mild cognitive impairment, and 136 Alzheimer’s disease; 72 ± 8 years; 543 women), with follow-up data available for 643 (915 ± 431 days from baseline). Three distinct spatiotemporal patterns were identified, primarily affecting the parietal, frontotemporal, and occipital lobes, respectively, in the early stages. The amyloid deposition rates differed between the subtypes, even after age, diagnosis, apolipoprotein E ε4 carriership (APOE), baseline amyloid burden, and tracer types were controlled for (occipital vs. parietal: *β* = 32.6, *P* < .001; occipital vs. frontotemporal: *β* = 17.0, *P* = .017; parietal vs. frontotemporal: *β* = 15.6, *P* = .026). The rates of change in cognitive scores, adjusted for age, diagnosis, APOE, baseline amyloid burden, baseline cognitive score, and tracer types also differed between the subtypes (occipital vs. Stage 0: *β* = 0.162, *P* = .021; occipital vs. parietal: *β* = 0.134, *P* = .013).

**Conclusion:**

Amyloid PET subtyping may serve as a valuable independent prognostic biomarker.

## Introduction

Alzheimer’s disease (AD), the leading form of dementia [1], places a substantial burden on healthcare due to its progressive nature and lack of effective treatments. This burden is projected to increase due to the combined effects of population growth and aging worldwide [2]. Recent advancements in anti-amyloid therapies [3, 4], despite demonstrating limited efficacy, offer a potential avenue for AD treatment by targeting the amyloid-beta protein. Amyloid PET plays a crucial role in quantifying the amyloid burden, enabling an early and independent diagnosis of AD [5]. Unlike cerebrospinal fluid or plasma biomarkers, amyloid PET offers high quantitative accuracy and spatial information, indicating the stages of the disease throughout its continuum [5]. To leverage these advantages, several studies have investigated the spatiotemporal patterns of amyloid deposition. While these studies, assuming a uniform pattern, have generally corroborated Braak et al.’s classic pathological findings [6], they have also highlighted the prognostic value of amyloid pathology for characterizing the risk of cognitive decline at the population level [7–9]. However, individual patterns can vary. Moreover, although a recent study identified amyloid deposition patterns [10], their direct links to cognitive decline have not been established.

Longitudinal pattern analyses typically require longitudinal datasets, which can be difficult to obtain from large populations. To address this limitation, a novel data-driven method called Subtype and Stage Inference (SuStaIn) [11] was recently developed. SuStaIn infers both the stages and subtypes of a progressive disease from heterogeneous cross-sectional data. A study of brain atrophy in AD has demonstrated its effectiveness in improving the prediction of the transition from mild cognitive impairment (MCI) to AD [11]. SuStaIn has also been used to identify the spatiotemporal phenotypes of tau accumulation, which have been associated with different clinical profiles [12], and to compare the amyloid and tau deposition patterns in AD [13].

This study aimed to examine the individual variability in amyloid deposition to identify the subtypes of its progression and investigate their potential to predict patient outcomes. To this end, SuStaIn was used to analyze a dataset provided by the Alzheimer’s Disease Neuroimaging Initiative (ADNI; http://adni.loni.usc.edu), one of the largest publicly available cohorts for AD research. As a technical refinement, the spatiotemporal patterns of amyloid deposition were extracted and summarized using independent component analysis (ICA) and were then fed into the SuStaIn model. The estimated subtypes were subsequently validated using follow-up amyloid PET data and longitudinal neuropsychological assessments to evaluate their clinical significance.

## Methods

### Participants

Individuals who participated in the ADNI 2, 3, or 4 study and underwent either [^18^F]florbetapir (FBP) or [^18^F]florbetaben (FBB) PET scans in conjunction with an MRI examination were included in the analysis. ADNI was launched in 2003 as a public-private partnership, led by Principal Investigator Michael W. Weiner, MD. The primary goal of ADNI has been to test whether serial MRI, PET, other biological markers, and clinical and neuropsychological assessment can be combined to measure the progression of MCI and early AD. This resulted in a cohort of 1,049 participants (466 cognitively normal; 162 early MCI; 102 late MCI; 183 MCI, and 136 AD). No data were excluded. The clinical characteristics of the ADNI cohort have been reported previously [14]. The diagnosis of AD was based on the criteria of the National Institute of Neurological and Communicative Disorders and Stroke and the Alzheimer’s Disease and Related Disorders Association [15]. Individuals with AD dementia were required to have a Mini-Mental StateExamination (MMSE) score [16] < 25 and a Clinical Dementia Rating Scale (CDR) scale score [17] > 0.5 at baseline. Individuals with MCI had memory issues with no significant functional impairment (MMSE scale > 23), a CDR-SB score of 0.5, and objective memory impairment determined using the Wechsler Memory Scale–Logical Memory II test. The cognitively normal had MMSE scores of > 24 and a global CDR score of 0 and did not meet the criteria for MCI and AD [18].

### Data preprocessing

*We downloaded the fully preprocessed amyloid PET images from the ADNI database. This processing pipeline includes frame-to-frame realignment for motion correction*,* averaging of frames into a single image*,* reorientation to a standard AC-PC alignment*,* and smoothing to a uniform isotropic resolution of 6 mm full-width at half-maximum.* The data were corregistered to T1-weighted MR images and normalized into standard Montreal Neurological Institute space using SPM12 (https://www.fil.ion.ucl.ac.uk/spm/software/spm12/). The data were converted into standard uptake value ratios (SUVR) relative to the whole cerebellum. To compensate for the tracer type differences, the data were further converted to Centiloid using Royse et al.’s equations [19] as follows:

$$FBP:\;Centiloid=188.22\:\times\:SUVR_{FBP}-189.16$$

$$FBB:\;Centiloid\;=\:157.15\:\times\:SUVRF_{FBB}-151.87$$

A voxel-based spatial ICA blinded to patient status, was performed to identify sources of amyloid deposition variation. *It is well-established that for imaging modalities with limited spatial resolution such as PET*,* the separation of gray matter signal from white matter and cerebrospinal fluid noise based solely on anatomical co-registration is imperfect due to significant partial volume effects* [20]. *To address this challenge and analyze the data with minimal a priori assumptions*,* we employed ICA on the whole brain data—a common methodology in neuroimaging that allows for the data-driven separation of biological patterns from structured noise* [21, 22], *with established applications in PET data analysis* [23, 24]. The Centiloid data were decomposed into 20 components using FastICA [25] within the whole brain mask, which was prepared as the intersection (> 50% of the participants) of each participant’s mask, including the voxels with a > 20% probability of being gray matter, white matter, or cerebrospinal fluid. *The model order was chosen in line with common practice in seminal neuroimaging studies that have successfully identified large-scale brain networks*,* as it provides a robust balance between capturing biological variance and avoiding model overfitting* [26, 27]. For each IC, significantly contributing voxels were identified by scaling each IC map to Z-scores and using Z = 1.96 as a threshold [22]. *All components were visually inspected in multiple planes*,* and* ten ICs, predominantly composed of gray matter, were selected as regions of interest (ROIs). These ROIs were *intersected with a grey matter mask* used to compute average Centiloid values, which were then fed into subtype and stage inference analyses.

### SuStaIn

The Z-score SuStaIn implementation [11] in pySuStaIn [28] (https://github.com/ucl-pond/pySuStaIn), cloned from the master branch on March 16, 2024, was used in Python 3.7. SuStaIn is a probabilistic machine learning algorithm that infers patterns of disease progression (subtypes) and an individual’s disease stage (i.e., the degree of progression within a subtype) [11]. The model uses a data likelihood based on the degree to which a biomarker measurement (in this study, the average IC ROI Centiloid values) deviates from normality to group events based on their associated Z-scores (1, 2, or 3 standard deviations away from the control population mean) [12, 13].

Before the SuStaIn analysis, each measure was adjusted for age and gender effects using a region-specific regression model, with the measure in question used as the dependent variable and age and sex used as the independent variables. *To isolate pathological changes from normal demographic variability*,* this model was fitted on the control group data alone to establish a healthy baseline. It was then applied to the entire cohort to calculate the residual (the deviation from this healthy baseline) for each participant.* The control group consisted of cognitively normal individuals who were negative for both apolipoprotein E (APOE) ε4 and amyloid. Amyloid negativity was determined using a UC Berkeley cortical summary SUVR analysis normalized to the whole cerebellum reference region, with thresholds of 1.11 [29] and 1.08 [19] for FBP and FBB, respectively. The regional measures were then residualized, and regional score probability matrices were constructed.

The optimal number of subtypes was determined by referring to the ten-fold cross-validation information criterion. Moreover, using positional variance diagram data, Pearson correlation coefficients were calculated to assess the similarity between the regional ordering of a single-type model and each subtype in the selected model, as well as between the subtypes themselves.

### Subtype analyses

Subtype differences were assessed independently of stage. Participants classified as Stage 0 were labeled as no subtype and included in the subsequent analysis. Multinomial logistic regression (MLR) was used to determine the effects of demographics and risk factors (age, sex, diagnostic group, APOE ε4 carriership, MMSE score, CDR-SB score, 13-item cognitive subscale of the Alzheimer’s Disease Assessment Scale [ADAS-Cog13] [30, 31] scores, and average whole-brain amyloid Centiloid value) and tracer type as a possible confounding factor on subtype assignments. A Kruskal-Wallis test was conducted to compare the assigned stages across the subtypes.

### Longitudinal validation

The optimal SuStaIn model derived from the baseline data was applied to longitudinal amyloid PET scans available for 643 participants (a mean of 915 ± 431 days from the baseline) to evaluate group categorization and stage inference stability. MLRs were performed to assess whether the amyloid deposition, MMSE, and ADAS-Cog13 change rates differed between subtypes, controlling for demographics and significant risk factors identified in the previous MLR analysis.

### Statistical Analyses

The statistical analyses were performed using SPSS 28.0.1.0 with a significance level of *P* <.05.

## Results

### Spatial ICA of amyloid PET data

**Fig. 1:**
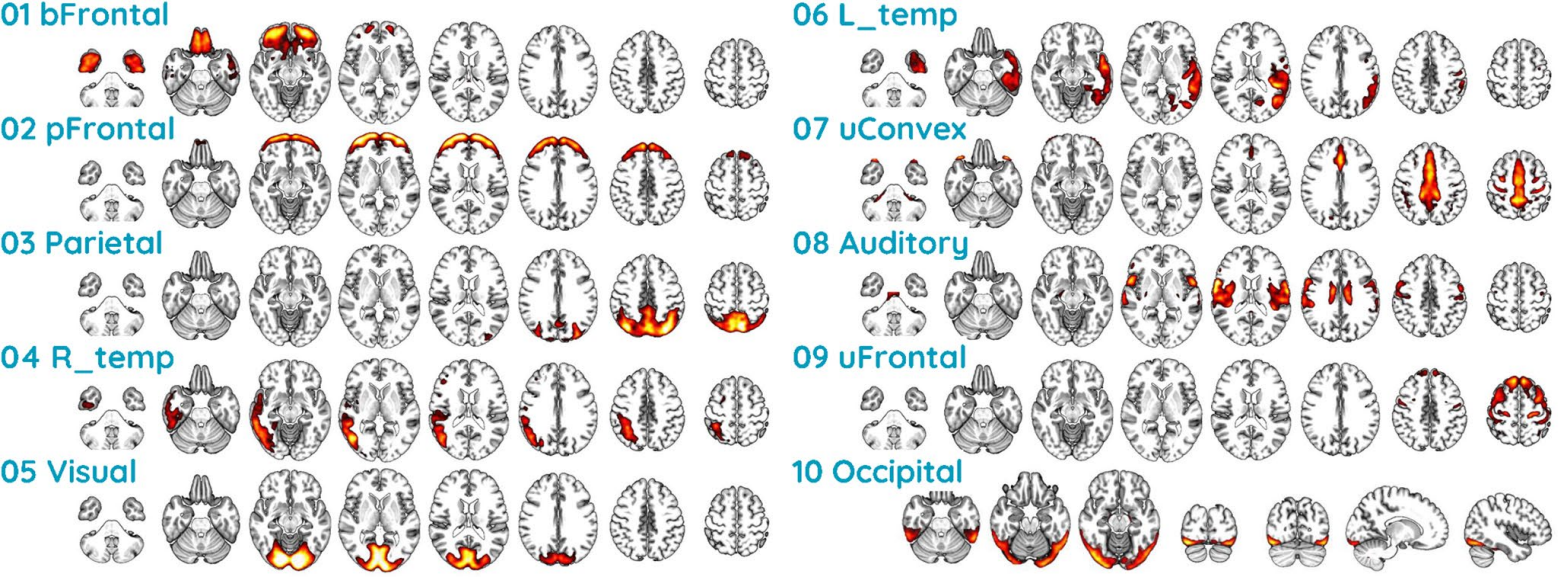
Independent Components of Spatially Coherent Amyloid Deposition Ten independent components predominantly composed of gray matter corresponding to distinct brain regions in (1) basal frontotemporal (bFrontal), (2) prefrontal (pFrontal), (3) parietal (precuneus), (4) right temporal (R_temp), (5) visual, (6) left temporal (L_temp), (7) upper convexity (superomedial frontoparietal or sensorimotor, uConvex), (8) auditory and sensorimotor, (9) upper prefrontal (uFrontal), and (10) occipital cortex were identified

The spatial ICA of the entire baseline amyloid PET dataset indicated 10 gray matter ICs in addition to those of white matter and cerebrospinal fluid. Each IC represented spatially coherent regions exhibiting covariation in amyloid deposition across participants. The gray matter ICs, depicted in Fig. 1, corresponded to distinct brain regions in (1) the basal frontotemporal, (2) prefrontal, (3) parietal (precuneus), (4) right temporal, (5) visual, (6) left temporal, (7) upper convexity (superomedial frontoparietal or sensorimotor), (8) auditory and sensorimotor, (9) upper prefrontal, and (10) occipital cortex.

### SuStaIn model selection and subtype assignment

**Fig. 2:**
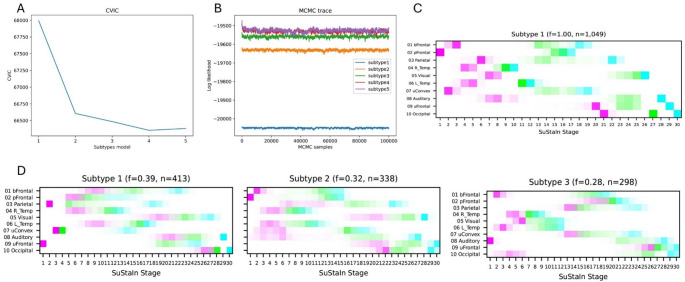
Cross Validation Results and Positional Variance Diagram of Amyloid Deposition Progression Identified by SuStaIn The results of a ten-fold cross-validation showed an improved model fit from one to four subtypes, as illustrated by (**A**) a decrease in the cross-validation information criterion (CVIC) and (**B**) the log-likelihood across 100,000 Markov chain Monte Carlo (MCMC) iterations. The diagrams illustrate the estimated patterns of amyloid deposition progression in the (**C**) single-type and (**D**) three-subtype models. Each color represents a different level of amyloid deposition severity (magenta for Z = 1, green for Z = 2, cyan for Z = 3), while the color intensity at each stage indicates the frequency with which each Z-score appears in that stage in the MCMC samples representing a potential model (the Z-scores in each sample are integers ranging from 0 to a maximum Z-score, defined as 5 in this study). The maps provide a visual representation of the variance of biomarkers across subtypes while accounting for the MCMC-based positional uncertainty estimates. bFrontal = basal frontal, L = left, pFrontal = prefrontal, R = right, SuStaIn = Subtype and Stage Inference, Temp = temporal, uFrontal = upper frontal, uConvex = upper convexity

**Fig. 3:**
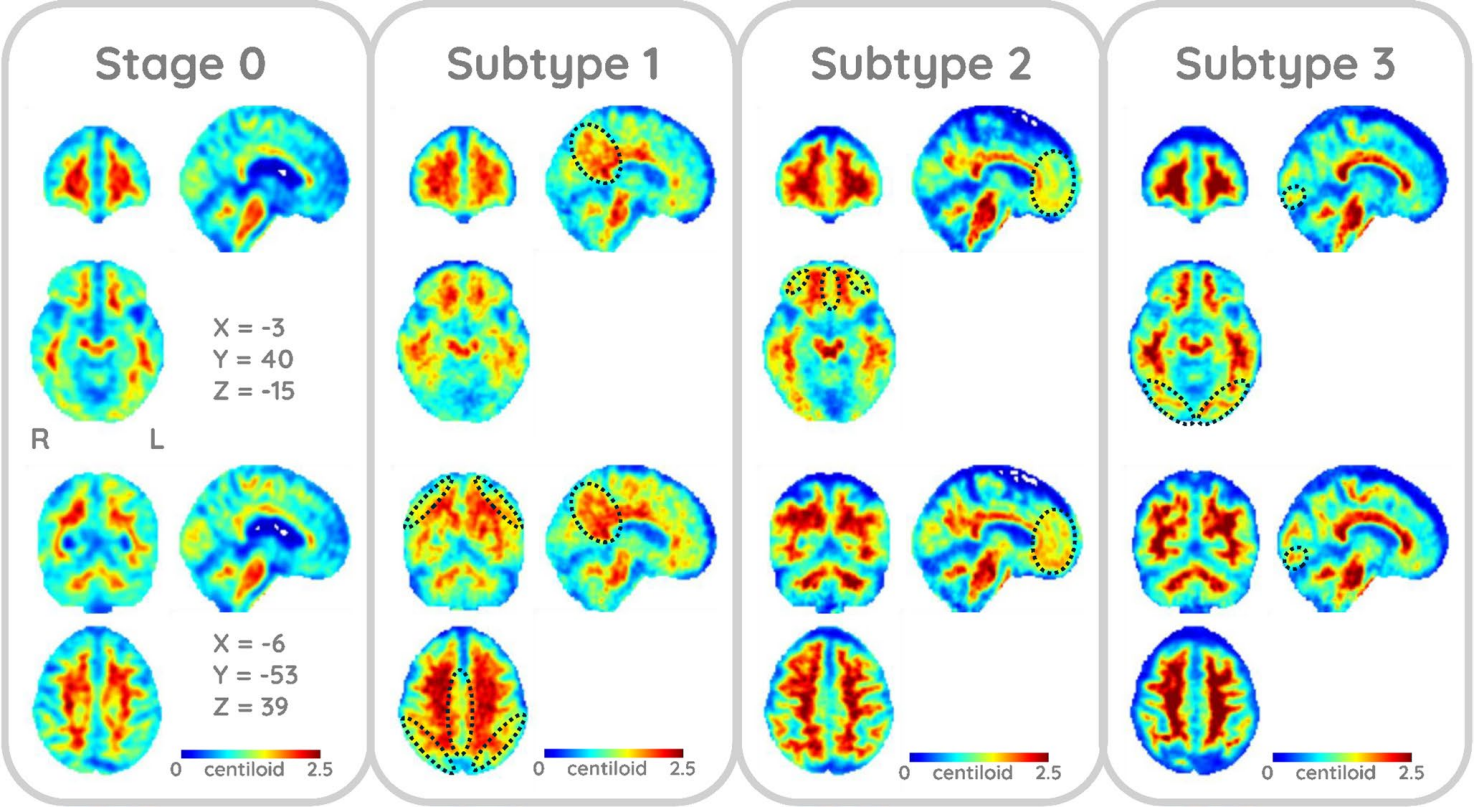
Example Cases Assigned to Each Subtype Stage 0: 80-year-old woman with mild cognitive impairment classified as Stage 0. Subtype 1: 80-year-old man with mild cognitive impairment classified as Subtype 1, Stage 2. Subtype 2: 90-year-old woman without cognitive impairment classified as Subtype 1, Stage 2. Subtype 3: 71-year-old woman with mild cognitive impairment classified as Subtype 3, Stage 2. Areas with enhanced gray matter amyloid deposition are indicated by dotted ellipses (Subtype 1: parietal lobes; Subtype 2: basal frontal and temporal lobes; Subtype 3: occipital cortex). All data were spatially normalized to the standard Montreal Neurological Institute space and displayed using the same color scale to facilitate comparisons

**Fig. 4:**
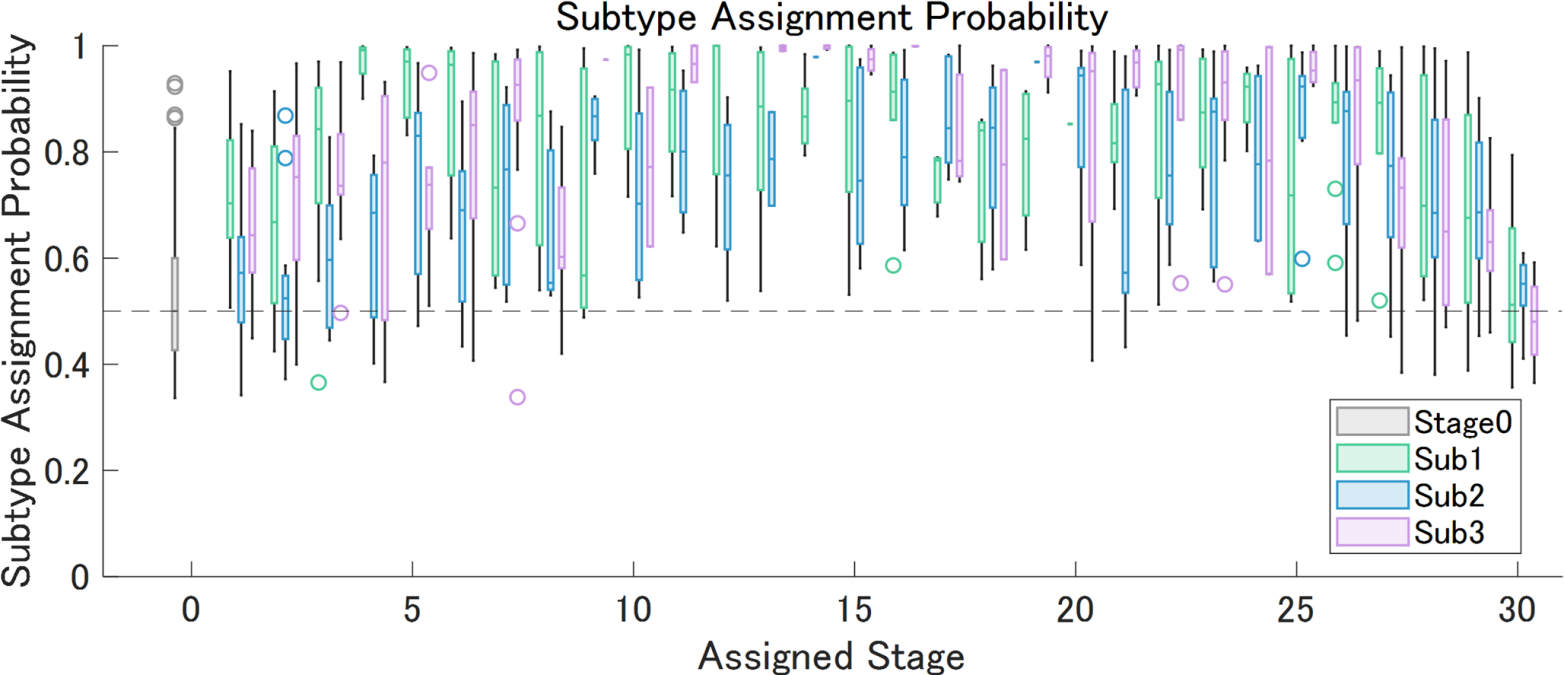
Subtype Assignment Probability Against Assigned Stage The box plots illustrate the relationship between stage assignment (x-axis) and the probability of subtype assignment (y-axis) for the baseline dataset. The probability tended to be lowest in stages closer to 0 or 30, in which subtype variance was likely minimized. Sub = subtype

The ten-fold cross-validation information criterion analysis indicated four distinct subtypes. However, due to their similarity and the small size of one subtype, a three-subtype model was ultimately chosen (Fig. 2A). The log-likelihood plot of each subtypes model on the test set suggests that no additional information is gained after 3 subtypes (Fig. 2B) also supports the choice of 3 subtypes. Of the 1,049 participants, 251, 235, and 197 were classified as Subtypes 1, 2, and 3, respectively, while 366 were classified as normal (Stage 0; Fig. 2D; Table 1). As illustrated in the positional variance diagrams [32] in Figs. 2C and D, the single-type amyloid deposition model closely resembled previous findings, with deposition beginning in the basal frontotemporal and prefrontal cortices (IC 01, 02), subsequently spreading to the parietotemporal lobes (IC 04, 06, 07), eventually reaching the visual cortex and precuneus (IC 05, 03), and finally affecting the sensorimotor and occipital cortices (IC 08, 09, 10; Figs. 2 and 3). Subtype 1 was characterized by early onset in the parietal including the precuneus (IC 03) and superomedial sensorimotor cortex (IC 07), and the upper prefrontal cortex (IC 09). Subtype 2 followed a pattern similar to that in the single-type model, with initial involvement of the basal frontotemporal and prefrontal cortices (IC 01, 02), followed by the parietotemporal lobes (IC 04, 06, 07), and so on. Subtype 3 was characterized by earlier onset and progression in the auditory (IC 08) and occipital cortex (IC 05, 10). The probability of subtype assignment tended to be lowest in stages closer to 0 or 30, where subtype variance was likely minimized (Fig. 4).

**Table 1:** Demographics and baseline risk factors for each subtype

Subtype	Diagnosis, *n* (%)			Age (yrs.), Mean ± SD	Female, %	APOE ε4, Mean ± SD	MMSE, Mean ± SD	ADAS-Cog13, Mean ± SD	Amyloid (CL), Mean ± SD
	CN	MCI	AD						
Stage 0 (*n* = 366)	208 (57)	145 (40)	13 (4)	71.0 ± 7.6	52.2	0.23 ± 0.46	28.7 ± 1.7	10.8 ± 6.8	−1.28 ± 14.1
Subtype 1 (*n* = 251)	91 (36)	108 (43)	52 (21)	74.1 ± 7.4	51.4	0.62 ± 0.67	27 ± 3.0	16.6 ± 10.4	57.7 ± 45.8
Subtype 2 (*n* = 235)	77 (33)	123 (52)	35 (15)	72.5 ± 7.4	53.2	0.81 ± 0.69	27.3 ± 2.9	15.8 ± 10.1	60.62 ± 39.1
Subtype 3 (*n* = 197)	90 (46)	71 (36)	36 (18)	71.9 ± 7.2	49.7	0.52 ± 0.68	27.6 ± 2.9	15.3 ± 9.9	43.24 ± 47.4
AD = Alzheimer’s disease, ADAS-Cog13 = 13-item cognitive subscale of the Alzheimer’s Disease Assessment Scale, APOE = apolipoprotein E, CL = Centiloid. CN = cognitively normal, MCI = mild cognitive impairment, MMSE = Mini Mental State Examination, SD = standard deviation, yrs. = years.

The correlations between the progression patterns of the single type and subtypes 1–3, computed using the positional variance diagram matrix, were 0.10, 0.32, and 0.23, respectively (*P* <.001). In terms of correlations between subtypes, the patterns of Subtypes 2 and 3 (*r* =.37, *P* <.001) showed a relatively strong correlation, while those of Subtypes 1 and 2 (*r* =.02, *P* >.05) and Subtypes 1 and 3 (*r* =.002, *P* >.05) showed weak correlations.

### Subtype differences

**Fig. 5:**
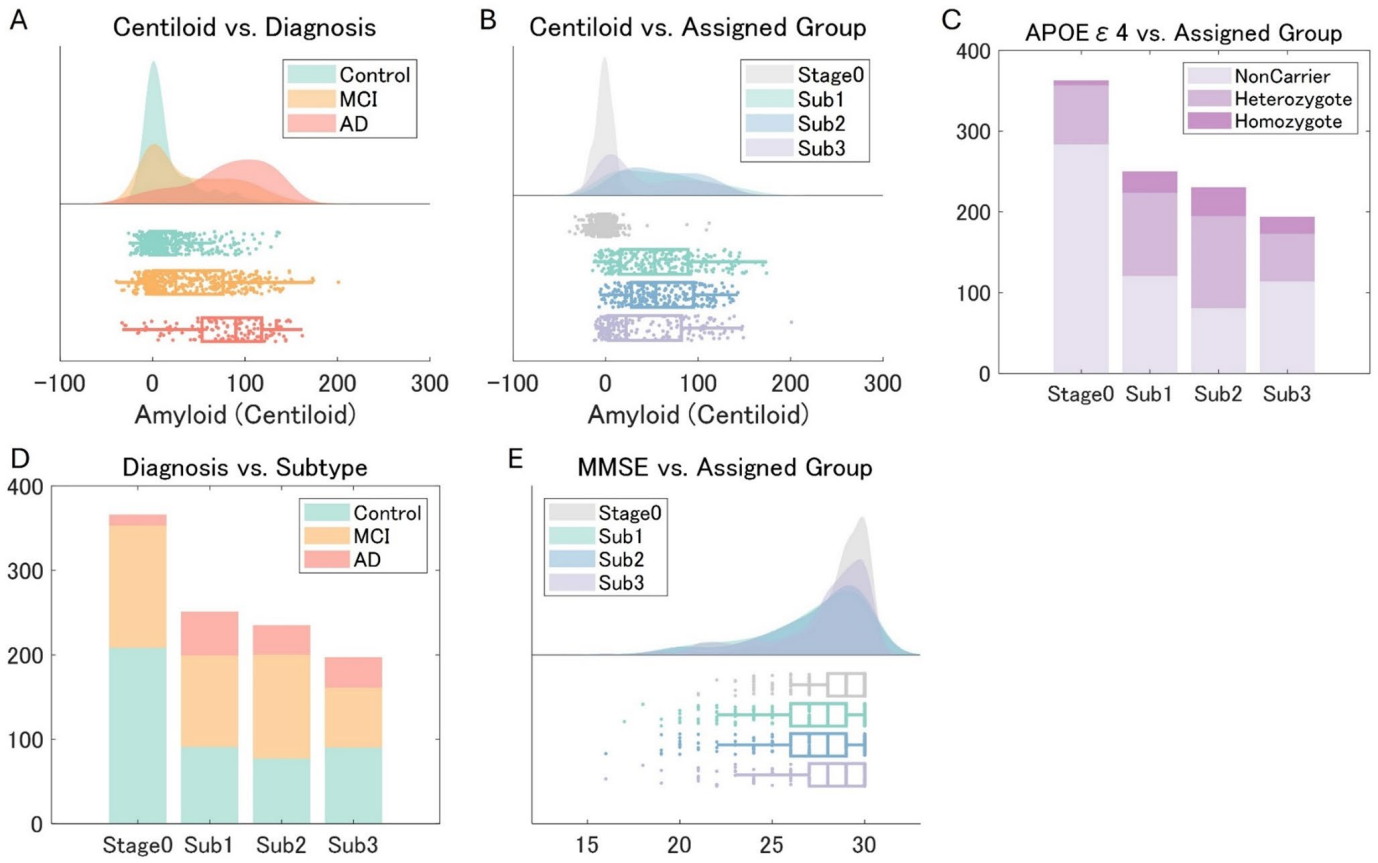
Diagnosis, Amyloid Deposition, APOE Profile and Subtypes The figures illustrate the relationships between (**A**) diagnosis and amyloid deposition, (**B**) subtypes and amyloid deposition, (**C**) apolipoprotein E ε4 carriership and subtypes, (**D**) diagnosis and subtypes, and (**E**) Mini Mental State Examination scores and subtypes. AD = Alzheimer’s disease, APOE = apolipoprotein E, MCI = mild cognitive impairment, MMSE = Mini Mental State, Sub = subtype

The demographic characteristics of the participants in each subtype are shown in Table 1; Fig. 5. The gender distribution was similar across subtypes. The participants in Subtype 1 were older than those in Subtype 3 (*β* = 0.049, *P* <.001). Subtype 2 had a higher proportion of MCI patients than Subtype 3 (*β* = 1.33, *P* =.005). The mean number of APOE ε4 copies was greater in Subtype 2 than in Subtypes 1 and 3 (vs. Subtype 1: *β* = 0.392, *P* =.009; vs. Subtype 3, *β* = 0.551, *P* <.001). The baseline MMSE, CDR-SB, and ADAS-Cog13 scores did not differ between the subtypes. The average whole-brain Centiloid value was significantly higher in Subtype 2 than in Stage 0 and Subtype 3 (vs. Stage 0: *β* = 0.115, *P* <.001; vs. Subtype 3: *β* = 0.007, *P* =.012). It was also higher in Subtype 1 and 3 than in Stage 0 (vs. Subtype 1: *β* = 0.113, *P* <.001; vs. Subtype 3: *β* = 0.108, *P* <.001). The rate of FBB usage was lower in Subtype 3 than the others (vs. Stage 0: *β* = 2.684, *P* <.001; vs. Subtype 1: *β* = 1.349, *P* = < 0.001; vs. Subtype 2: *β* = 0.851, *P* =.003). A Kruskal-Wallis test indicated a significant stage assignment difference between the three subtypes, χ²(2) = 6.92, *P* =.031, for Subtype 1 (Median = 13), Subtype 2 (Median = 16), and Subtype 3 (Median = 8), with a significant pairwise difference between Subtype 2 and 3 (*P* =.01).

### Longitudinal validation

**Fig. 6:**
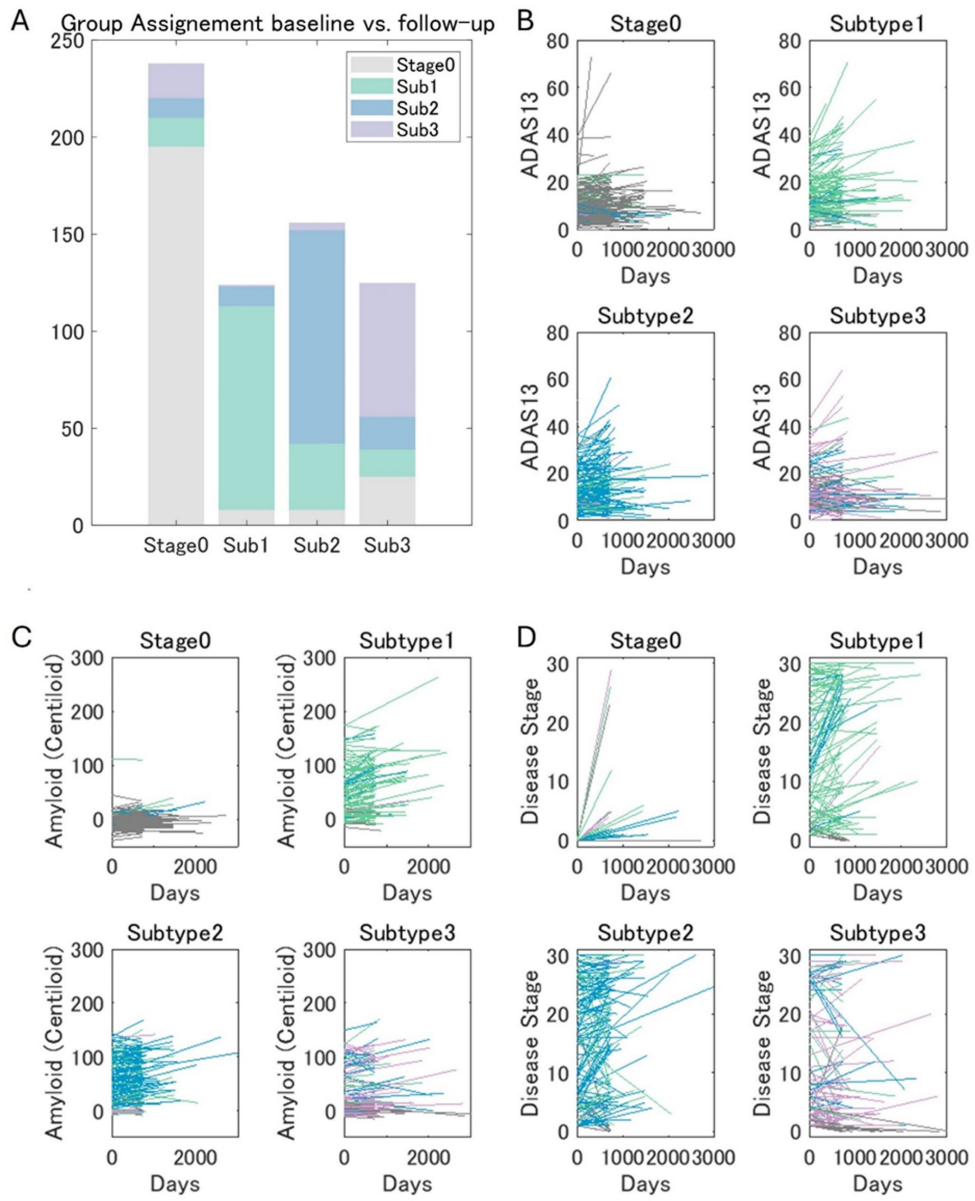
Longitudinal Evaluation (**A**) Subtype assignment at baseline vs. follow-up. Longitudinal changes from baseline in (**B**) ADAS-Cog13 score, (**C**) amyloid accumulation (in Centiloid), and (**D**) assigned disease stages in each group. The line colors represent the subtype assignment at follow-up. ADAS-Cog13 = 13-item cognitive subscale of the Alzheimer’s Disease Assessment Scale, Sub = subtype

Compared to the baseline data, the follow-up (longitudinal) amyloid PET data included fewer AD patients (*χ*^2^ = 19.73, *P* <.001). Of the 479 (74.5%) participants who maintained their subtype, 195 were in Stage 0, 105 in Subtype 1, 110 in Subtype 2, and 69 in Subtype 3. Forty-three (6.7%) progressed from Stage 0 to a higher stage. The subtypes of 80 (12.4%) participants changed at follow-up, while 8 (1.2%) Subtype 1, 8 (1.2%) Subtype 2, and 25 (3.9%) Subtype 3 changed to Stage 0, respectively. An examination of stage assignment stability independently of subtypes showed that 252 (39.2%) participants remained in the same stage at follow-up, 247 (38.4%) progressed to a later stage, and 144 (22.4%) regressed to an earlier stage (Fig. 6). Among those who progressed to a higher stage, Subtype 3 assignments at follow-up were significantly less frequent compared to the baseline (Subtype, 1 40.5%; Subtype 2, 40.5%; Subtype 3, 19.0%; *χ*^2^ = 9.19, *P* =.01).

The amyloid deposition rates differed between the subtypes assigned at baseline even after age, diagnosis, APOE ε4 carriership, baseline amyloid burden, and tracer types were accounted for (Fig. 6). Specifically, the longitudinal accumulation rates were lower in Subtype 3 than in Subtypes 1 and 2 (vs. Subtype 1: *β* = 32.6, *P* <.001; vs. Subtype 2: *β* = 17.0, *P* =.017), lower in Stage 0 than in Subtype 1 and Subtype 2 (vs. Subtype 1; *β* = 51.9, *P* <.001; vs. Subtype 2: *β* = 36.3, *P* =.018), and higher in Subtype 1 than in Subtype 2 (*β* = 15.6, *P* =.026). The rate of change in ADAS-Cog13 scores after age, diagnosis, APOE ε4 carriership, baseline amyloid burden, baseline ADAS-Cog13, and tracer type were controlled for was also significantly lower in Subtype 3 than in Stage 0 and Subtype 1 (vs. Stage 0: *β* = 0.162, *P* =.021; vs. Subtype 1: *β* = 0.134, *P* =.013).

## Discussion

Despite the ongoing debate—*fueled by both its weak correlation with cognitive symptoms relative to tau pathology and the inconclusive outcomes of many past anti-amyloid therapies* [33, 34]—the amyloid cascade hypothesis, which posits amyloid-beta aggregation as a key AD pathological hallmark [35], *remains the central framework guiding the development of anti-amyloid therapies*. *The recent approval of anti-amyloid drugs has*,* therefore*,* has intensified the need to understand how amyloid patterns*,* not just overall burden*,* correlate with cognitive decline*. *Against this backdrop*, this study investigated the heterogeneity of amyloid deposition patterns in AD using a data-driven subtyping and staging model (SuStaIn). *SuStaIn is uniquely suited for this purpose as it overcomes the critical limitations of simpler modeling approaches when applied to heterogeneous populations. Whereas traditional “stages-only” models assume a single disease trajectory for all individuals*,* potentially missing distinct biological pathways*,* “subtypes-only” models like clustering assume all participants are at a comparable disease stage*,* which is invalid for a large cohort study spanning the full clinical spectrum like ADNI. SuStaIn is specifically designed to disentangle these two sources of heterogeneity by simultaneously resolving both phenotypic subtypes and their distinct temporal progression patterns from cross-sectional data alone.*

Our analysis of cross-sectional amyloid PET data from 1,049 participants indicated three distinct subtypes. Classification stability was assessed using follow-up data from 634 participants, of whom 75% remained in the same category, while 7% progressed from Stage 0 to a higher stage. Notably, while baseline cognitive function did not differ between the subtypes, the rate of cognitive decline varied significantly, even when other risk and confounding factors were accounted for. This demonstrates that the spatial pattern of amyloid pathology holds significant prognostic value beyond what is captured by global burden alone.

Among the three subtypes, Subtype 2 was more similar to the typical progression pattern of amyloid deposition, which is characterized by early involvement of basal frontotemporal areas. This subtype was also associated with a higher proportion of APOE ε4 carriership and greater global amyloid accumulation. In contrast, Subtype 3, characterized by early amyloid deposition in the auditory and occipital cortices, showed a lower total amyloid burden and a lower prevalence of MCI than the other subtypes. Although Subtype 1, characterized by early deposition in the parietal lobes was associated with a greater mean age and faster longitudinal amyloid accumulation, there were no significant differences in mean age between Subtypes 2, 3 and Stage 0.

Despite differences in datasets and analytical strategies, the subtype identification in this study showed some similarities with a previous study that also identified “parietal” and “occipital” subtypes in addition to the “frontal (standard)” pattern of amyloid deposition [10], which would correspond to Subtypes 1, 3, and 2, respectively, in this study. Both studies observed a higher global amyloid burden and a higher rate of APOE ε4 carriership in the standard subtype than in the other subtypes. However, the two studies’ results differed somewhat in terms of subtype assignment and the relationships between subtypes and risk factors. Specifically, most participants (52.5%) in the previous study were assigned to the standard pattern, whereas the distribution was more even in this study. Moreover, the longitudinal amyloid accumulation rates were higher in the occipital type in the previous study, while they were lower in Subtype 3 in this study. The discrepancies in age, baseline amyloid deposition, and proportion of cognitively unimpaired participants between the two studies may have contributed to the differences in subtype distribution. The lower mean age (68.7 ± 9.1 years), lower baseline amyloid deposition (24.2 ± 35.8 CL), and higher prevalence of cognitively unimpaired individuals (63%) in the previous study compared to this study (72.2 ± 7.5 years, 35.1 ± 45.6 CL, and 44%, respectively) may have obscured subtype differences. The results of the two studies should be interpreted and compared by considering these factors. Nevertheless, it might also be possible that the ICA-based measurement of amyloid burden, rather than a reliance on predefined ROIs, was more sensitive to subtle differences in spatiotemporal patterns. This approach, which defines the target variables for the SuStaIn analysis, offers an objective and unbiased representation of amyloid deposition by considering covariation patterns across participants. Ten gray matter ICs, analogous to the “spatial networks” commonly investigated in functional brain imaging studies, were extracted using spatial ICA. These components exhibited a symmetrical distribution across the brain, except for the temporal lobe components, which were divided into two. This division aligns with previous pathological study findings [6] and is assumed to have enabled a more granular assessment of amyloid deposition progression. As for longitudinal study, we also note that the subjects included in the previous longitudinal study (376 from ADNI + 143 from OASIS) are substantially different in the present study’s 643 subjects from ADNI.

In either case, a key finding of this study was that the rate of cognitive decline varied significantly between the subtypes, even when other risk and confounding factors were accounted for, suggesting that, unlike the differences described above, the observed differences in cognitive decline rate were not due to possible confounding factors (i.e., differences in the distribution of the baseline factors). These findings, in concert with those of the previous study [10], *add a clinically relevant dimension to the interpretation of amyloid PET by showing that the topography of the pathology*,* not just its overall burden*,* has prognostic significance. This is particularly important when considering the distinct roles of the core Alzheimer’s pathologies. It is a cornerstone of neuropathology that the burden and distribution of tau strongly correlate with the severity of concurrent cognitive impairment* [36–40]. *In contrast*,* amyloid-β deposition is a much earlier pathological event*,* often preceding significant tau pathology and cognitive symptoms by many years*,* as formalized in the AT(N) framework* [5, 41]. *Our results bridge this temporal gap*,* showing that the spatial pattern of this early-appearing amyloid pathology holds independent predictive power for the disease’s future clinical course. The immediate relevance of this finding is underscored by the current clinical landscape*,* where amyloid PET is now an indispensable and reimbursed tool for determining eligibility for anti-amyloid treatments*,* whereas tau PET remains largely a research modality.*

There are some limitations and technical considerations to be taken into account when interpreting the results. First, while the longitudinal subtype assignments were relatively stable, the test–retest reliability of the classification in shorter intervals could not be assessed. Second, the data was derived from 57 facilities participating in the ADNI consortium. This multi-site approach enhances the generalizability of our findings, mitigating potential biases often associated with single-center studies. Nevertheless, the inclusion of data from two different tracer types presents a limitation. *Specifically*,* the harmonization between tracers relied on a single*,* global conversion equation for all ICA-derived ROIs*,* a methodological choice that may not perfectly account for tracer-specific regional binding properties* [7]. *This could have biased the classification of patients*,* such as* leading to an underestimation of subtype 3 in the FBB group. It should also be noted that part of the ADNI data was also included in the previous study [10]. Finally, the mechanisms giving rise to the different spatial patterns remain unclear. *Third*,* several limitations inherent to SuStaIn itself*,* as described by its developers* [11], *warrant consideration. A key caveat is that SuStaIn is designed to estimate a set of distinct trajectories*,* which may be a useful simplification of what is potentially a continuous spectrum of disease progression patterns in the underlying biology. Another assumption of the model is that biomarker variance is independent*,* whereas in reality*,* biomarkers often co-vary due to shared biological processes. Although SuStaIn has been shown to be robust to this*,* our results could be influenced by these unmodeled covariances. Moreover*,* while our study demonstrates relatively stable subtype assignments*,* the model’s ability to assign a specific subtype is less certain at the very earliest and latest disease stages*,* where biomarker patterns are either minimal or uniformly widespread.*

In conclusion, building upon the established foundation provided by previous studies, our subtype and stage inference analysis of amyloid PET data revealed replicable spatiotemporal patterns in amyloid deposition, which can serve as independent prognostic biomarkers of AD stages. While further validation and investigations of the underlying pathology are required, this study demonstrates the potential of amyloid PET subtyping to improve patient selection for anti-amyloid therapies and enhance our understanding of the pathophysiology of AD.

## Data Availability

No datasets were generated or analysed during the current study.
